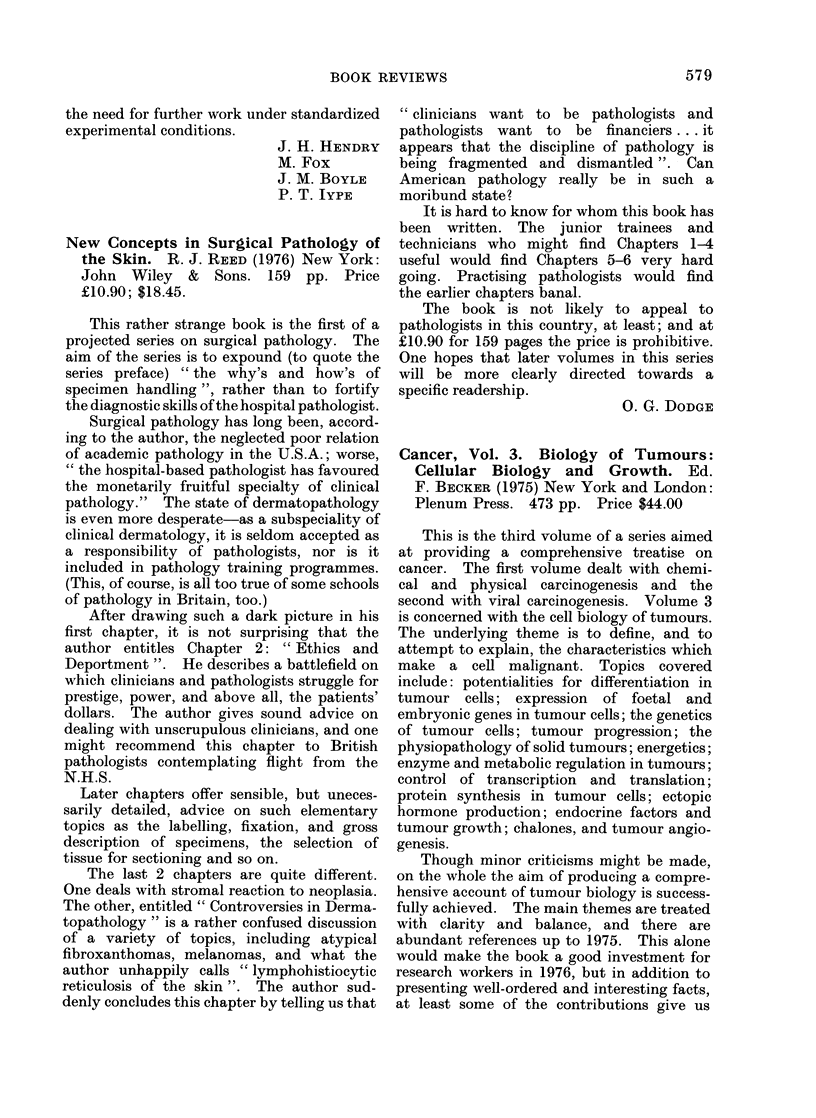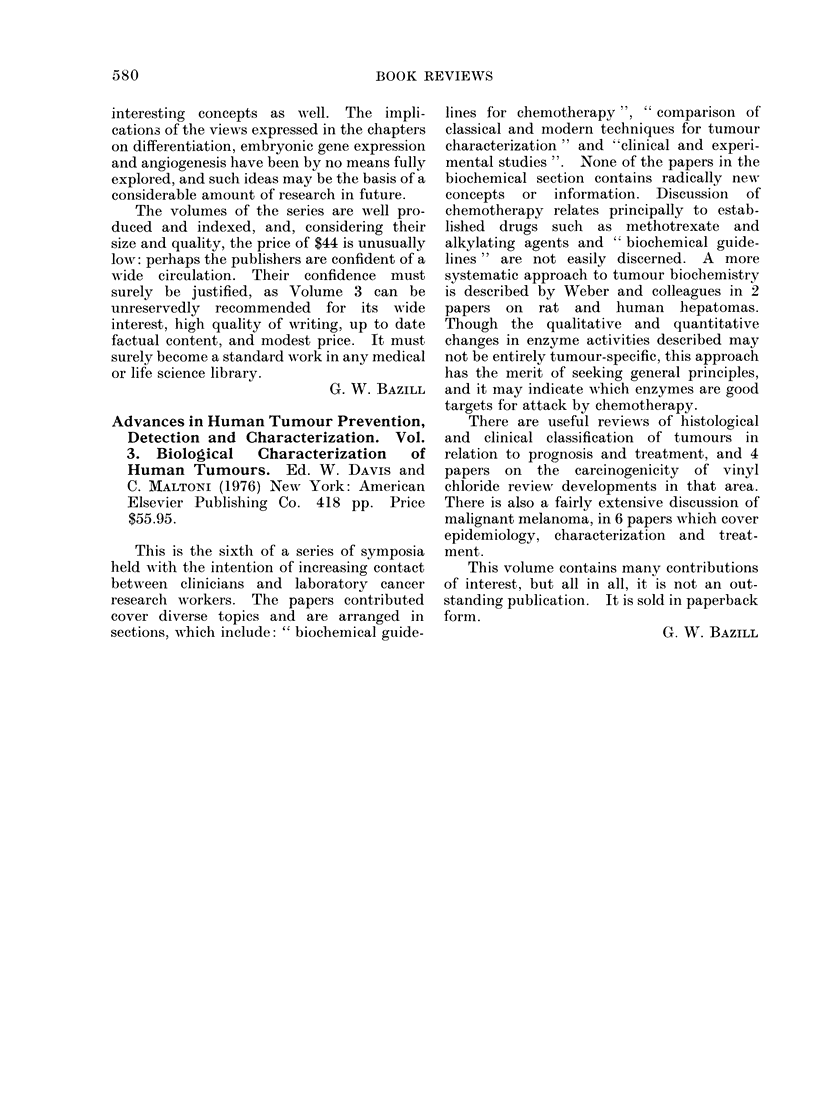# Cancer, Vol. 3. Biology of Tumours: Cellular Biology and Growth

**Published:** 1976-11

**Authors:** G. W. Bazill


**Cancer, Vol. 3. Biology of Tumours: Cellular Biology and Growth.** Ed. F. BECKER (1975) New York and London: Plenum Press. 473 pp. Price $44.00

This is the third volume of a series aimed at providing a comprehensive treatise on cancer. The first volume dealt with chemical and physical carcinogenesis and the second with viral carcinogenesis. Volume 3 is concerned with the cell biology of tumours. The underlying theme is to define, and to attempt to explain, the characteristics which make a cell malignant. Topics covered include: potentialities for differentiation in tumour cells; expression of foetal and embryonic genes in tumour cells; the genetics of tumour cells; tumour progression; the physiopathology of solid tumours; energetics; enzyme and metabolic regulation in tumours; control of transcription and translation; protein synthesis in tumour cells; ectopic hormone production; endocrine factors and tumour growth; chalones, and tumour angiogenesis.

Though minor criticisms might be made, on the whole the aim of producing a comprehensive account of tumour biology is successfully achieved. The main themes are treated with clarity and balance, and there are abundant references up to 1975. This alone would make the book a good investment for research workers in 1976, but in addition to presenting well-ordered and interesting facts, at least some of the contributions give us interesting concepts as well. The implications of the views expressed in the chapters on differentiation, embryonic gene expression and angiogenesis have been by no means fully explored, and such ideas may be the basis of a considerable amount of research in future.

The volumes of the series are well produced and indexed, and, considering their size and quality, the price of $44 is unusually low: perhaps the publishers are confident of a wide circulation. Their confidence must surely be justified, as Volume 3 can be unreservedly recommended for its wide interest, high quality of writing, up to date factual content, and modest price. It must surely become a standard work in any medical or life science library.

G. W. Bazill